# The prevalence and clinical significance of intracranial vertebral artery terminated in posterior inferior cerebellar artery: A multicenter hospital-based study in China

**DOI:** 10.3389/fneur.2022.1026614

**Published:** 2022-11-08

**Authors:** Juehua Zhu, Ruiyun Huang, Kaiwen Ye, Hongbing Chen, Zheng Dai, Yongjun Jiang

**Affiliations:** ^1^Department of Neurology, The First Affiliated Hospital of Soochow University, Suzhou, China; ^2^Department of Neurology, The Second Affiliated Hospital of Guangzhou Medical University, Guangzhou, China; ^3^Department of Neurology and Stroke Center, The First Affiliated Hospital, Sun Yat-sen University, Guangzhou, China; ^4^Department of Neurology, Wuxi People's Hospital, Wuxi, China

**Keywords:** intracranial vertebral artery, posterior inferior cerebellar artery, stenosis, stroke, intracranial vertebral artery terminated in posterior inferior cerebellar artery

## Abstract

**Objective:**

Intracranial vertebral artery terminated in the posterior inferior cerebellar artery (PICA-VA) is the most popular variant of the posterior inferior cerebellar artery, while its prevalence and clinical significance remained unclear. In the present study, we aimed to investigate the prevalence and clinical significance of PICA-VA.

**Methods:**

This was a multicenter hospital-based cross-sectional study. Patients were enrolled for cerebral MRI and MRA within 1 week of stroke onset. Clinical characteristics were recorded. PICA-VA is termed as a vertebral artery that does not communicate with the basilar artery but terminates in an ipsilateral PICA. We observed the prevalence of PICA-VA and identified a relationship between PICA-VA and vertebrobasilar stroke.

**Results:**

From 1 August 2015 to 31 May 2017, a total of 2,528 patients were enrolled in the present study. Among them, 95 patients (3.76%, 95/2,528) had the variation of PICA-VA, 51 of which (53.7%) were located on the right side. The prevalence of vertebrobasilar stroke was considerably higher in patients with PICA-VA than those without (40.2%, 37/92 vs. 17.1%, 417/2,436, *p* < 0.01). PICA-VA was an independent risk for vertebrobasilar stroke after being adjusted for a history of intracranial hemorrhage, diabetes, body mass index, and triglyceride.

**Conclusion:**

The present study showed that 3.76% of patients with acute stroke had PICA-VA, which independently increased the risk of acute vertebrobasilar stroke.

## Introduction

The vertebral arteries (VAs) originate from the subclavian artery, and the two VAs converge to form the BA at the lower margin of the junction of the medulla oblongata and the pons. The diameters of both VAs are generally asymmetrical. Vertebral artery dominance (VAD) is an extremely common variation of VAs. Although without a standard definition, VAD refers to the obvious asymmetry of the vertebral artery diameters on both sides (1). In most conditions, if a VA union occurs, the non-dominant VA is likely to obtain sufficient blood supply from the dominant one. Thus, traditionally VADs have traditionally been regarded as a normal congenital vascular variant of VAs. In recent years, evidence has been raised that VAD is an independent risk factor for posterior circulation ischemic stroke (1–4).

The posterior inferior cerebellar artery (PICA) is a principal branch of the VA and has no alternative collateral circulation (5). One less common variation of VA is that a hypoplastic VA does not unite with the contralateral VA but ends into the PICA. This type of variation is termed a vertebral artery terminated in PICA (PICA-VA) (6–8). It was also referred to as “basilarization” one of the ICVA by Caplan (9).

Although the existence of PICA-VA is known, relatively little information has been revealed from clinical studies regarding its prevalence and relationship to vertebrobasilar stroke. One previous duplex sonography and magnetic resonance angiography (MRA) based study that included 80 patients and 80 controls suggested that the prevalence of PICA-VA was much higher (18.7 vs. 6.3%) in patients with a history of vertebrobasilar stroke or transient ischemic attack (TIA) than that in the matched normal controls (7). More recently, MRA screening for evidence of stroke before cervical spine surgery demonstrated that PICA-VA was more frequent in patients aged older than 50 years than those younger than 39 years (13.3 vs. 1.6%) (10).

The detection of PICA-VA relies on the development of cerebrovascular imaging modalities, samples, and subject heterogeneity. Digital subtraction angiography (DSA) is supposed to be a more precise technique to identify PICA-VA. Osborn et al. found that about 0.1% of patients had the variation by DSA (11). However, numerous patients did not receive invasive DSA, and this might have led to an underestimation of the prevalence of PICA-VA. Non-invasive methods like magnetic resonance angiography (MRA) have been used to evaluate the cerebral artery. In a small cohort, ~1 in ten patients (11.7%, 15/128) had PICA-VA (6).

In the present study, we aimed to investigate the prevalence of PICA-VA in a multicenter hospital-based cross-sectional study with a larger sample size and explore the relationship between PICA-VA and vertebrobasilar stroke.

## Methods

### Population

A retrospective review of a prospectively complied multicenter hospital-based computerized database was performed (12). The study was approved by the local institutional ethics committee. Informed consent was signed by the patient or an authorized person.

### Experimental design

**Figure 1:**
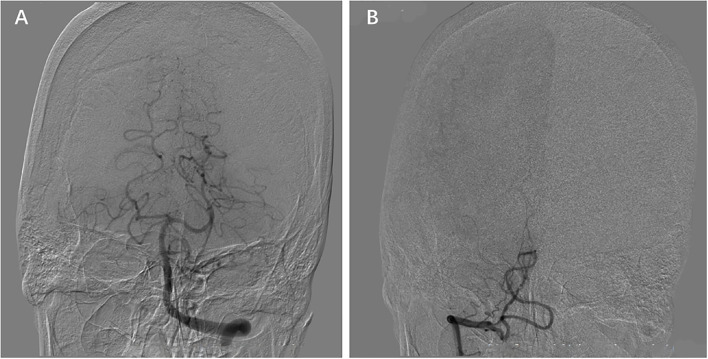
Posterior inferior cerebellar artery (PICA) termination of the vertebral artery (PICA-VA). The VA on one side does not join the contralateral VA **(B)**, instead terminating as the posterior inferior cerebellar artery **(A)**.

From 1 August 2015 to 31 May 2017, patients from Jinling Hospital, The Second Affiliated Hospital of Guangzhou Medical University, The First Affiliated Hospital of Soochow University, and Wuxi People's Hospital were screened. The patient who met the following criteria was included in the present study: (1) More than 18 years old. (2) Stroke was defined by brain magnetic resonance imaging (MRI), and acute neurological deficits evaluated by neurologists were required; (3) Cerebral MRI including T1, T2, and DWI images and MRA were performed within 1 week of stroke onset; (4) Intracranial VA and PICA were clearly visible; (5) Cervical carotid artery and vertebral artery were detected by ultrasound, CTA, MRA, or DSA. PICA-VA was confirmed by the DSA; and (6) Clinical information was available. The patient who had one of the following criteria was excluded from the analysis: (1) Occlusion of vertebral artery or basilar artery; patients were also included in the analysis when occluded vessels were recanalized. (2) Unique vertebral artery. (3) Hemorrhagic stroke. The hemorrhagic transformation within an ischemic infarction was also included in the analysis. (4) Spinal infarction. (5) Infarction distributed both in the anterior and posterior circulation areas. PICA-VA was defined as VA not communicating with the basilar artery but ending into the ipsilateral PICA according to MRA and verified by DSA (Figure 1).

### Digital subtraction angiography imaging analysis

Digital Subtraction Angiography was performed with femoral catheterization by using the Seldinger technique with a biplane DSA unit with rotational capabilities (Axiom Artis dTA; Siemens Healthcare). Typically, 6–9 ml of non-ionic contrast medium (iopromide, 300 mg of iodine per milliliter, Ultravist 300; Bayer Schering) was used per acquisition, usually consisting of one anteroposterior, one lateral, and one or two oblique views. With the catheter in each of the three major arteries (both internal carotid arteries and one or more vertebral arteries), standard anteroposterior, lateral, and oblique DSA images were obtained.

### Data collection

Trained interviewers conducted face-to-face interviews at the hospital to collect data at baseline. On admission, demographic information (including age and gender), risk factors for stroke [including body mass index (BMI), current smoking, hypertension, diabetes, previous stroke or transient ischemic attack (TIA) or intracranial hemorrhage (ICH), previous coronary artery disease (CHD), and atrial fibrillation], and laboratory examinations [including total cholesterol, TG, HDL cholesterol, LDL cholesterol, Glomerular Filtration Rate (GFR)], and current medications (including antihypertensive agents, lipid-lowering agents, antiplatelet agents, and anticoagulant agents) on admission were acquired. Current drinking was defined as heavy intake (≥14 drinks/week in women or 21 drinks/week in men) or episodic heavy intake (≥5 drinks/episode at least once per month) (13). Diabetes was defined as A1C ≥6.5%, FBG levels ≥7.0 mmol/L, blood glucose ≥11.1 mmol/L at 2 h by oral glucose tolerance test (OGTT), blood glucose ≥11.1 mmol/L at random glucose test, any use of glucose-lowing drugs, or any self-reported history of diabetes (14). Hypertension was defined as systolic pressure ≥140 mmHg or diastolic pressure ≥90 mmHg, any use of antihypertensive drugs, or a self-reported history of hypertension (15).

### Statistical analysis

Statistical analysis was performed using SPSS for Windows, version 22.0 (SPSS Inc., Chicago, IL). All parametric results are expressed as mean ± SD or percentage (for discrete variables). The Chi-square test was used for the comparison of categorical variables, and all the baseline variables were included in the logistic regression multivariable model. Two-sided *p* < 0.05 was considered significant. To adjust for confounding factors with *p* < 0.10, multivariable logistic regression analysis was used to assess any independent factors of vertebrobasilar stroke.

## Results

### Incidence of PICA-VA in vertebrobasilar stroke

From 1 August 2015 to 31 May 2017, a total of 2,528 patients were enrolled in the present study. The patient demographics are presented in Table 1. In 431 of the 2,528 patients, infarcts were identified in the territory of the posterior circulation, including the brain stem, cerebellum, occipital cortex, temporal cortex, and thalamus. Ninety-five (3.76%, 95/2,528) patients had the variation of PICA-VA, 51 of which (53.7%, 51/95) were located on the right side. Patients with PICA-VA presented a higher incidence of vertebrobasilar stroke (42.1, vs. 16.1%, *p* < 0.01) and a higher incidence of contralateral intracranial VA stenosis (33.7 vs. 17.5%, *p* < 0.01). Patients with PICA-VA demonstrated a different pattern of infarction distribution relative to those without PICA-CA. Infarction of patients with PICA-VA was most frequently located in the cerebellum (45.0%), followed by the pontine (30.0%) and medulla (10.0%). For patients without PICA-VA, the top 3 locations were pontine (44.2%), cerebellum (33.5%), and midbrain (11.0%).

**Table 1:** Characteristics of stroke patients with or without PICA-VA.

**Characteristics**	**Patients with PICA-VA** **(*n* = 95)**	**Patients without PICA-VA** **(*n* = 2,433)**	***p*-value**
Gender (male)	59 (62.1%)	1,459 (60.0%)	0.68
Age (yrs)	65.48 ± 12.10	65.82 ± 11.84	0.79
BMI	26.91 ± 3.21	26.44 ± 4.02	0.26
Hypertension	73 (76.8%)	1,797 (73.8%)	0.52
Hyperlipidemia			
TC (mM)	4.32 ± 1.25	4.31 ± 1.24	0.97
LDL (mM)	2.72 ± 1.06	2.64 ± 1.07	0.99
HDL (mM)	0.89 ± 0.13	0.91 ± 0.13	0.45
TG (mM)	2.76 ± 0.40	2.73 ± 0.40	0.19
Diabetes	20 (21.0%)	398 (16.3%)	0.23
Current smoker	15 (15.8%)	437 (18.0%)	0.59
Drinker	9 (9.5%)	288 (11.8%)	0.48
GFR (ml/min)	102.95 ± 11.13	102.21 ± 10.43	0.50
Stroke history			
Stroke or TIA	5 (5.3%)	96 (3.9%)	0.79
ICH	3 (3.2%)	81 (3.3%)	0.93
CHD	9 (9.5%)	230 (9.4%)	0.99
Atrial fibrillation	19 (20.0%)	530 (21.8%)	0.68
Vertebrobasilar stroke	40 (42.1%)	391 (16.1%)	< 0.01
Midbrain	3 (7.5%)	43 (11.0%)	
Pontine	12 (30.0%)	173 (44.2%)	
Medulla	4 (10.0%)	27 (6.9%)	
Cerebellum	18 (45.0%)	131 (33.5%)	
Multiple	3 (7.5%)	17 (4.3%)	
Intracranial VA stenosis	32 (33.7%)	426 (17.5%)	<0.01

VA, vertebral artery; PICA, posterior inferior cerebellar artery; PICA-VA, PICA termination of the VA; BMI, body mass index; TC, total cholesterol; LDL, low-density lipoprotein; HDL, high-density lipoprotein; TG, triglyceride; HCY, homocysteine; GFR, glomerular filtration rate; TIA, transient ischemic attack; ICH, intracranial hemorrhage; CHD, coronal heart disease; VA, vertebral artery.

### PICA-VA increased risk of vertebrobasilar stroke

As shown in Table 2, compared with those without vertebrobasilar stroke, patients with vertebrobasilar stroke presented a higher incidence of PICA-VA (9.3 vs. 2.6%, *p* < 0.01) as well as higher body mass index (BMI, 27.12 ± 5.83 vs. 26.33 ± 3.48, *p* < 0.01), higher level of triglyceride (2.78 ± 0.49 mmol/L vs. 2.72 ± 0.38 mmol/L, *p* < 0.01), and higher rate of intracranial hemorrhage history (5.3 vs. 2.9%, *p* = 0.01).

**Table 2:** Characteristics of stroke patients with or without posterior circulation stroke.

**Characteristics**	**Vertebrobasilar stroke** **(*n* = 431)**	**Without vertebrobasilar stroke** **(*n* = 2,097)**	***P*-value**
Gender (male)	254 (58.9%)	1264 (60.2%)	0.60
Age (yrs)	65.52 ± 12.30	65.86 ± 11.86	0.66
BMI	27.12 ± 5.83	26.33 ± 3.48	< 0.01
Hypertension	326 (75.6%)	1,544 (73.6%)	0.39
Hyperlipidemia			
TC (mM)	4.11 ± 2.55	4.13 ± 2.65	0.87
LDL (mM)	2.61 ± 1.32	2.74 ± 0.97	0.89
HDL (mM)	0.89 ± 0.13	0.91 ± 0.13	0.11
TG (mM)	2.78 ± 0.49	2.72 ± 0.38	< 0.01
Diabetes	59 (13.7%)	359 (17.1%)	0.08
Current smoker	73 (16.9%)	379 (18.0%)	0.58
GFR (ml/min)	101.83 ± 10.40	102.33 ± 10.47	0.36
Drinker	46 (10.7%)	251 (12.0%)	0.45
Stroke history			
Stroke or TIA	23(5.3%)	78 (3.7%)	0.25
ICH	23 (5.3%)	61 (2.9%)	0.01
CHD	48 (11.1%)	191 (9.1%)	0.19
Atrial fibrillation	88 (20.4%)	461 (22.0%)	0.47
PICA-VA	40 (9.3%)	55 (2.6%)	<0.01

VA, vertebral artery; PICA, posterior inferior cerebellar artery; PICA-VA, PICA termination of the VA; TC, total cholesterol; LDL, low-density lipoprotein; HDL, high-density lipoprotein; TG, triglyceride; HCY, homocysteine; GFR, glomerular filtration rate; TIA, transient ischemic attack; ICH, intracranial hemorrhage; CHD, coronal heart disease.

### PICA-VA was independently correlated with vertebrobasilar stroke

In multivariate analysis for vertebrobasilar stroke as summarized in Table 3, PICA-VA was independently associated with a higher rate of vertebrobasilar stroke with an adjusted OR of 3.842 (95% CI = 2.513–5.874, *p* < 0.01). In addition, intracranial hemorrhage history (OR_adjust_ = 1.934, 95% CI = 1.174–3.187, *p* = 0.01) and higher BMI (OR_adjust_ = 1.045, 95% CI = 1.01–1.081, *p* = 0.011) remained significant risk factor.

**Table 3:** Multivariate logistic regression of risk factors of vertebrobasilar stroke.

	**B**	**S.E**.	**Wald**	***p***	**Exp(B)**	**95% CI**	
PICA-VA	1.346	0.217	38.63	< 0.01	3.842	2.513	5.874
ICH	0.66	0.255	6.708	0.01	1.934	1.174	3.187
BMI	0.044	0.017	6.394	0.011	1.045	1.01	1.081
TG	0.089	0.173	0.265	0.607	1.093	0.778	1.536
Diabetes	−0.295	0.154	3.653	0.056	0.745	0.55	1.008

VA, vertebral artery; PICA, posterior inferior cerebellar artery; PICA-VA, PICA termination of the VA; BMI, body mass index; TG, triglyceride; ICH, intracranial hemorrhage.

## Discussion

The present study was a multicenter hospital-based cross-sectional study. Our data showed that 3.76% of acute stroke patients had a PICA-VA variant. PICA-VA was associated with the elevated incidence of vertebrobasilar stroke. The strength of the present study was that we demonstrated the prevalence of PICA-VA and identified its clinical significance in a relatively large sample.

The vertebrobasilar system supplies the brainstem, cerebellum, and part of the cortex. Variations were relatively common in the vertebrobasilar systems for 8–20.2% of hypoplastic VAs were observed (16). As for the intracranial part of VAs, the two intracranial VAs were usually asymmetrical and one might be 2–3 times the size of the remaining one (17). In some cases, the distal segment of intracranial VA from the origin of PICA to BA was absent and intracranial VA seemed to end in PICA in the angiogram (18), which was defined as PICA-VA (19). The prevalence of PICA-VA in different studies remained controversial. In the early time, DSA was used to determine the presence of PICA-VA. Osborn et al. found that only 0.07% of patients had this variation, and Greenberg et al. showed that 2% of the normal population had PICA-VA (20). Given the potential risk of DSA, many patients did not receive the DSA, which might have underdetermined the prevalence of PICA-VA. Liu et al. showed that 6.3% of PICA-VA in the normal population and 19% in patients with stroke, were higher than that of our study (7). In the present study, 3.76% of all patients with acute stroke and 9.28% of patients with vertebrobasilar stroke had a variation of PICA-VA. The differences may be due to the number of samples and the method used to detect the PICA-VA. Liu et al. only recruited 80 patients, while we included 2,528 patients. Moreover, Liu et al. used MRA to detect PICA-VA only. This may overestimate the prevalence because it was difficult to distinguish some asymptomatic isolated V4 occlusion from the PICA-VA by MRA. We screened the PICA-VA by MRA and then confirmed it with DSA. Our finding was partly consistent with a systematic review, which showed that 7.7% of vertebrobasilar stroke patients had PICA-VA (21).

The posterior inferior cerebellar artery had been recognized as the normal variation of intracranial VA (20). However, we found that PICA-VA was an independent risk factor for vertebrobasilar stroke. For patients with vertebrobasilar stroke, the variation was increased by about 2-fold times when compared to that without vertebrobasilar stroke (9.28 vs. 3.76%). This was the first study to demonstrate the clinical significance of PICA-VA. Moreover, we also showed there was the distribution of infarcts was different between patients with and without PICA-VA. Pontine infarction was the most abundant in patients without PICA-VA. For patients with PICA-VA, most of the infarcts occurred in the cerebellum, followed by the pontine. For the PICA-VA, the embolism would only occlude the PICA when the embolism goes through the ipsilateral VA. This might cause extra infarction in the cerebellum. For the general patients with vertebrobasilar stroke, pontine infarction was the dominant (14, 22).

The underlying mechanism of PICA-VA-induced vertebrobasilar stroke was still unknown. In the previous study, patients with PICA-VA were more prone to develop atherosclerosis than those without PICA-VA (23). VA diameter of the PICA-VA was significantly smaller, which was thought of as VA hypoplasia (6). The reason might be that there was higher asymmetry and resistance index, and lower mean flow and end-diastolic velocity in PICA-VA (7). Reduction in vertebral flow volume has been recognized as a predisposing factor for vertebrobasilar stroke (3). The high risk of atherosclerosis might increase the prevalence of stroke onset.

There were some limitations of the present study. First, the study was hospital based. The subjects in the present study had an acute stroke within 1 week. Normal people or other patients with non-acute strokes were not included. This may lead to some bias. Second, the vertebrobasilar stroke was performed based on the DWI image. The patients with DWI negative stroke and TIA were identified as patients with non-infarction. This may underestimate the prevalence of stroke. Diagnosis of TIA relied on the quality and quantity of information available and the time of assessment (24). However, the symptoms in the posterior circulation like isolated vertigo were normal in other patients with non-stroke (25). New infarction in the brainstem was hard to diagnose based on CT or other image modalities. In this way, we used MRI for the diagnosis of vertebrobasilar stroke.

## Conclusion

In conclusion, our data showed that 3.76% of acute stroke patients had a PICA-VA variant, which was associated with a risk of vertebrobasilar stroke.

## Data availability statement

The original contributions presented in the study are included in the article/supplementary material, further inquiries can be directed to the corresponding author.

## Ethics statement

The studies involving human participants were reviewed and approved by Ethics Committee of the Second Affiliated Hospital of Guangzhou Medical University. The patients/participants provided their written informed consent to participate in this study.

## Author contributions

YJ: conceptualization. RH and KY: data collection. HC and ZD: investigation. JZ and YJ: formal analysis and writing. All authors contributed to the article and approved the submitted version.

## Funding

This study was financially supported by the National Science Foundation of China (81471181 and 81870933), the Opening Lab Program of Guangzhou Medical University (0506308), Guangdong Basic and Applied Basic Research Foundation (2021A1515011351), Guangzhou Science and Technology Project (202102010127 to YJ), National Natural Science Foundation of China (Grant No. 82171282), Suzhou Mingsheng Science Technology Project (Grant No. 2020101), Scientific Research Project of Jiangsu Provincial Health and Wellness Committee (Grant No. M2020064), and Health Youth Backbone Project of Suzhou City (Qngg2021003 to JZ).

## Conflict of interest

The authors declare that the research was conducted in the absence of any commercial or financial relationships that could be construed as a potential conflict of interest.

## Publisher's note

All claims expressed in this article are solely those of the authors and do not necessarily represent those of their affiliated organizations, or those of the publisher, the editors and the reviewers. Any product that may be evaluated in this article, or claim that may be made by its manufacturer, is not guaranteed or endorsed by the publisher.
